# Communicating the effectiveness and ineffectiveness of government policies and their impact on public support: a systematic review with meta-analysis

**DOI:** 10.1098/rsos.190522

**Published:** 2020-01-15

**Authors:** J. P. Reynolds, K. Stautz, M. Pilling, S. van der Linden, T. M. Marteau

**Affiliations:** 1 Behaviour and Health Research Unit, University of Cambridge, Cambridge, UK; 2 Department of Psychology, University of Cambridge, Cambridge, UK

**Keywords:** communication, policy, evidence, acceptability, attitudes, beliefs

## Abstract

Low public support for government interventions in health, environment and other policy domains can be a barrier to implementation. Communicating evidence of policy effectiveness has been used to influence attitudes towards policies, with mixed results. This review provides the first systematic synthesis of such studies. Eligible studies were randomized controlled experiments that included an intervention group that provided evidence of a policy's effectiveness or ineffectiveness at achieving a salient outcome, and measured policy support. From 6498 abstracts examined, there were 45 effect sizes from 36 eligible studies. In total, 35 (*N* = 30 858) communicated evidence of effectiveness, and 10 (*N* = 5078) communicated evidence of ineffectiveness. Random effects meta-analysis revealed that communicating evidence of a policy's effectiveness increased support for the policy (SMD = 0.11, 95% CI [0.07, 0.15], *p* < 0.0001), equivalent to support increasing from 50% to 54% (95% CI [53%, 56%]). Communicating evidence of ineffectiveness decreased policy support (SMD = −0.14, 95% CI [−0.22, −0.06], *p* < 0.001), equivalent to support decreasing from 50% to 44% (95% CI [41%, 47%]). These findings suggest that public support for policies in a range of domains is sensitive to evidence of their effectiveness, as well as their ineffectiveness.

## 1. Introduction

Obesity rates are high and rising worldwide [1], human-caused climate change proceeds towards a level incompatible with life in a growing number of regions [2], and mass shootings occur almost daily in the USA [3]. These problems, among many others, require the action of policy makers, yet low public support can present a barrier for such action, especially in an increasingly complex and politicized information environment [4–7]. There have been many attempts to increase support for various policies across issue domains, and communicating evidence of a policy's effectiveness has potential as one possible strategy [8]. The current study aims to systematically synthesize the evidence for changing public attitudes and support for policies by communicating evidence about a policy's effectiveness at achieving its goal. This is the first study of which we are aware to conduct such a synthesis.

The perceived effectiveness of a policy at achieving its goal has consistently been found as one of the strongest predictors of support over a range of policies [8–14]. Reflecting these findings, a number of studies have communicated evidence that a policy is effective as an intervention to increase policy support, with mixed results. Studies that have communicated unconfounded evidence that a policy is effective have mostly found increased support for the policy, with perceived effectiveness acting as the mechanism behind this change [8,15–17]. Studies that have communicated messages containing evidence of effectiveness in addition to further information have yielded mixed results, with the mechanism(s) remaining unclear [18–21]. Further studies communicating evidence that a policy is ineffective or that it has undesirable outcomes, such as economic and health costs, have also led to mixed results [15,22,23]. Even within a single study, the same intervention has been shown to have different effects on different policies [18], leading to uncertainty about the effectiveness of such approaches.

This work can be understood more broadly in terms of how changes in relevant beliefs (e.g. the perceived effectiveness of a policy) can engender a change in basic attitudes (e.g. support for the policy; [24]). Public support can be defined as how individuals feel and think about the implementation of a policy. As such, this area of research sits within literatures in judgement and decision-making concerning the impact of information—including misinformation—on a broad range of psychological and behavioural outcomes, including voting and support for policies. Importantly, in a so-called post-truth society where the nature of evidence is increasingly contested [25], recent studies have been stimulated by a concern that providing factual information has little impact on people's beliefs and might sometimes even have the opposite of the intended effect, serving only to entrench pre-existing beliefs when these differ markedly from the evidence being presented. In general, there are three possible responses to being presented with evidence: maintenance of one's current beliefs, polarization of an existing belief away from beliefs consonant with the evidence, and updating of beliefs in a direction consistent with the evidence. Although people are generally motivated to hold accurate beliefs about the world [26], directionally biased assimilation can occur when people selectively credit or discredit ‘evidence’ to arrive at a preferred conclusion. The confirmation bias—the seeking and interpretation of evidence that is consistent with one's intial beliefs—has emerged as a central mechanism for understanding why people do not change their beliefs when provided with evidence [27]. In addition, sometimes people update their beliefs in the opposite direction of the evidence. For example, one influential study found that, when considering the effectiveness of capital punishment on crime prevention, exposure to mixed evidence caused students to strengthen their prior convictions [28]. This process is variously described as ‘belief polarization’, ‘boomerang' or ‘backfire effect' [29]. Subsequent studies, however, found that true belief polarization is not the norm, and in fact, a relatively rare phenomenon; ‘by and large, citizens heed factual information, even when such information challenges their ideological commitments' [30–32].

Indeed, the most likely response when confronted with disconfirming or contrary evidence is for the individual to move their belief toward the evidence [33–36]. This is often observed as a mean change on a response scale. This indicates that while people may report different numbers on a scale, they may not have shifted their beliefs categorically from one side to another, for example from disbelieving to believing in the existence of human-caused climate change. The number of people who change their position entirely is smaller than the number who alter specific beliefs [37,38]. At the same time, evidence-based opinion formation is hampered by the fact that in the current media environment, falsehoods can spread deeper and faster than factual information [39]. In addition, the advent of social media has enabled communities of like-minded people to easily share ideas that conform with and reinforce existing beliefs (i.e. echo chambers; [40,41]). One common example of echo-chambers are those organized around politics [42]. It is unsurprising then, that some beliefs and attitudes are harder to change than others, such as those that have strong political implications [29,35,43]. Attitudes towards the implementation of policies are one such area that may be harder to shift, especially given that public support for some policies is increasingly influenced by political identities [44], for example, around climate change, abortion, and gun control. Yet, even so, a large literature in persuasion psychology shows that the framing of information can significantly alter the value and weight people attach to their beliefs about policies and their effectiveness [45].

In other words, different ways of communicating the same information may be more or less successful at changing attitudes [46] and therefore need to be considered. For example, quantitative estimates of policy effectiveness are potentially more effective than qualitative estimates in allowing people to understand gradations of effectiveness [47,48]; e.g. the difference between very effective and extremely effective is unclear whereas the difference between 10% reduction in crime and 15% reduction in crime is clear. Despite this potential benefit in comprehension from quantifying effectiveness, it is not clear if this would translate into greater support for that policy. The use of uncertainty qualifiers has seen a surge of interest in recent years [49,50]. However, it remains unclear how the use of uncertainty qualifiers when describing policy effectiveness would affect attitudes towards the policy. Because people are generally averse to ambiguity [51], if the evidence about the effectiveness of the policy were presented as uncertain, this may mute any change in attitudes towards the implementation of that policy.

Using meta-analytic evidence synthesis, the aim was to assess whether communicating evidence that a policy is effective or ineffective changes support for the policy and if so, by how much. The authors hypothesize that both sets of evidence will change people's attitudes in the direction consistent with the evidence that they receive. Additionally, it is predicted that the effects will be larger when communicating ineffectiveness information due to negativity biases [52]. The moderating effects of policy domain and intervention characteristics were also tested.

## 2. Method

This systematic review is reported in line with PRISMA (preferred reporting items for systematic reviews and meta-analyses) guidelines [53]. The review protocol was prospectively registered in the PROSPERO database (ID: http://www.crd.york.ac.uk/PROSPERO/display_record.php?ID=CRD42017079524). The data and code are available at https://osf.io/4gjur/.

### 2.1. Eligibility criteria

Policies eligible for inclusion were actual or hypothetical policy interventions to tackle a problem that might be implemented by local or national governments, or by supranational bodies, such as the World Health Organization. Eligible studies include randomized experiments in which one group of participants received information about the (in)effectiveness or impact of at least one policy and a control group of participants that did not receive any information about the (in)effectiveness or impact of the policy. Within-participants, between-participants and quasi-experimental (i.e. experiments without random assignment) designs were also eligible.

There were no restrictions on types of participants. Eligible interventions for the evidence of effectiveness analysis were those that provided information regarding evidence of the impact of the policy, in terms of the potential benefits, or how the policy would address a specified problem (i.e. be effective). This includes evidence that discusses qualitative or quantitative descriptions of the magnitude of the effect, or assertions that describe the effect without referring to its magnitude.

Eligible interventions for the evidence of ineffectiveness analysis were those that provided information regarding evidence of the impact of the policy, in terms of the potential harms, or how the policy would fail to achieve its intended purposes (i.e. be ineffective). This includes evidence that discusses qualitative or quantitative descriptions of the magnitude of the effect, or describe the effect without referring to its magnitude. Both effectiveness and ineffectiveness interventions were eligible if they asserted that a policy was effective, even if they did not specify that the information originated from an expert or from research, e.g. ‘this tax would lead to a 1.6% reduction in obesity' [8]. Interventions were ineligible if the information was presented as coming from the general public or non-experts, e.g. ‘Some people believe that increasing trade with other nations creates jobs and allows you to buy goods and services at lower prices' [54].

Eligible comparators were control groups that received no information pertaining to the effectiveness or impact of the policy. Eligible outcomes were the acceptability of a specific policy or set of policies, defined as the level of support or attitude toward the implementation of policy/policies, measured using response scales that allow for a binary assessment (i.e. support or oppose), or a graded degree of support or opposition. Measures of support for public or societal action in general, not linked to a specific action or policy, were excluded [35].

### 2.2. Literature search

The search strategy was developed with the assistance of an information scientist. Nine electronic databases were searched: ASSIA, EconLit, EMBASE, Open Grey, PsycINFO, Public Affairs Information Service, PubMED, Science Direct, and Web of Science. There were no restrictions on language or publication date. Search terms were as follows:

(accept* OR support* OR favour* OR agree* OR attitude* OR opinion* OR perspective*) AND (policy OR regulation OR intervention OR proposal OR action OR government) AND experiment AND (information OR vignette OR narrative OR evidence OR statement OR frame OR framing) AND (effect* OR impact OR outcome OR consequence)

These terms were selected and developed based on terms that were used in the eligible papers that we had already located prior to the review. Database searches were completed up to October 2017. Two researchers (K.S. and J.P.R.) independently screened title-abstract records for eligibility. Screening discrepancies were resolved by discussion. Full texts of provisionally eligible records were retrieved via electronic library resources, screened independently by two researchers (K.S. and J.P.R.), and judged to be eligible or excluded with reasons recorded. Database searches were supplemented with snowball searches and forward citation tracking (using Google Scholar) of eligible articles, and reference list searches of relevant review articles. Requests for further unpublished data were made to the corresponding authors of eligible articles.

### 2.3. Data extraction

#### 2.3.1. Information extracted

One researcher (either K.S. or J.P.R.) extracted information from eligible studies. Once extraction was complete, each researcher cross-checked the extraction completed by the other. Discrepancies in extracted information were resolved by discussion and by consulting with a third or fourth researcher if necessary (T.M.M. or S.v.d.L.).

The following information was extracted from each study: author name, year of publication, country in which data were collected, setting, study design, sample characteristics (age, gender, ethnicity, socio-economic position), inclusion/exclusion criteria for participants, policy domain and proposed policy, details of the intervention(s) (using the below coding scheme), details of the information given to the control group, dependent variable(s), method of analysis, outcome data, and information needed for quality assessment.

#### 2.3.2. Coding

A coding scheme for interventions was developed prior to data extraction and conducted by two researchers in duplicate. Inter-rater agreement was high (93%). Discrepancies in extracted information were resolved by discussion and by consulting with a third or fourth researcher if necessary. Ten features of each intervention were coded: other information communicated (the nature of the problem being addressed by the policy, mechanism by which the policy affects the problem, defining/explaining concepts, inoculating against alternative policies, downsides to the proposed policy), content of message (quantitative statement of effect, qualitative statement of effect, assertion of effect; see electronic supplementary material, table S3 for examples), medium (e.g. text on screen, face to face interview), length of treatment, proportion of information that was a statement of effectiveness, readability (assessed using the Gunning-Fog Index), source of information used, attributed source of information, and stated level of uncertainty, if any.

#### 2.3.3. Missing data

Where original studies did not provide adequate data for use in meta-analysis or intervention coding, requests for further information were made to the corresponding authors of those studies. Of 24 authors contacted, 17 provided these requested data. The full text of the experimental interventions was unavailable for only one of the papers included in the meta-analyses and was therefore not coded. In the effectiveness meta-analysis, effect sizes and associated confidence intervals (CIs) were calculated from means, standard deviations and sample sizes (*k* = 30), *t* statistics and sample sizes (*k* = 4), and odds ratios and sample sizes (*k* = 1). This means that one of the 35 effect sizes in the evidence of effectiveness meta-analysis was converted from a dichotomous outcome ([55], formula 7.1). In one case [56], where only total sample size was available, it was assumed that the group sizes were equal. In the evidence of ineffectiveness meta-analysis, most effect sizes and associated confidence intervals were calculated using means, standard deviations and sample sizes (*k* = 9), and one was calculated using a *t* statistic and sample size.

### 2.4. Risk of bias

The quality assessment tool for quantitative studies [57] was used to provide a methodological rating for each eligible study on the following categories: selection bias, study design, blinding, data collection methods, and withdrawals and dropouts. One category—confounders—was not factored into the quality score following advice from a reviewer. Ratings on these categories were then used to create a summary rating for the study: weak, moderate or strong. These ratings were conducted by two separate researchers, with disagreements resolved by discussion and a third researcher in certain cases. The agreement between the two primary reviewers was substantial (linear weighted *κ* = 0.78). Sensitivity analyses were conducted to determine if the main results remained after only including studies that were not at high risk of bias. Funnel plots and Egger's regression were used to detect small study bias (funnel plot asymmetry), in which smaller studies have larger effect sizes. This can indicate publication bias or other forms of bias. Where these funnel plots suggested that bias was present, the trim and fill method was used to produce the best estimate of the unbiased effect size [55,58]. This approach identifies any potential funnel plot asymmetry and imputes ‘missing' studies which should remove the asymmetry.

### 2.5. Synthesis of results

Quantitative synthesis (meta-analysis) was used to calculate summary effect sizes. Our primary meta-analysis examined the effect of presenting information of effectiveness that was in favour of a policy, compared to no information, on public support. A secondary meta-analysis examined the effect on public support of presenting evidence of ineffectiveness and/or information that the policy had outcomes that were not in its favour when compared to no information.

As eligible studies used a range of different measures to assess public support, study-level standardized mean differences (SMDs; specifically, Hedges' *g*) were computed between comparison groups with 95% confidence intervals [55]. To ensure independence of observations in any meta-analysis, the following decision rules were followed prior to conducting the analyses: (i) in studies that included multiple eligible outcome measures, the combined means and variances were calculated using standard formulae ([55] equations 24.1 and 24.2); and (ii) when multiple interventions were eligible, the intervention containing evidence of effectiveness alone would be chosen over an intervention containing evidence of effectiveness *and* information on the nature of the problem. In cases where no intervention could be singled out using this method, the eligible intervention groups were combined into a single group as recommended by the Cochrane Handbook [58].

For each meta-analysis, random effects were used rather than fixed effects due to significant heterogeneity between studies. Statistical heterogeneity was assessed using the *χ^2^* test and the *I^2^* statistic. As stated in the review registration, follow-up analyses were conducted by policy domain (health, environment, other), and intervention characteristics: the readability of the interventions (assessed with the Gunning Fog index), by the presence of uncertainty in the evidence of effectiveness, and by how effectiveness is presented (assertions that the policy has a particular outcome versus descriptions of the magnitude by which the policy has a particular outcome). Meta-regressions were conducted to investigate these potential moderation effects. Sensitivity analyses were also conducted to determine if the main results remained unaltered after excluding studies that were at high risk of bias and also after excluding studies that included confounded interventions, e.g. interventions that included evidence of effectiveness plus other information thought to influence policy support. R v. 3.3.3 package metafor were used to run the meta-analyses and meta-regressions [59,60].

The pooled effect sizes and associated confidence intervals were converted to changes in policy support proportions using a number needed to treat formula (table 2; [61]). The R code used to estimate changes in policy support proportions can be found in the supplement.

## 3. Results

### 3.1. Study selection

**Figure 1.**
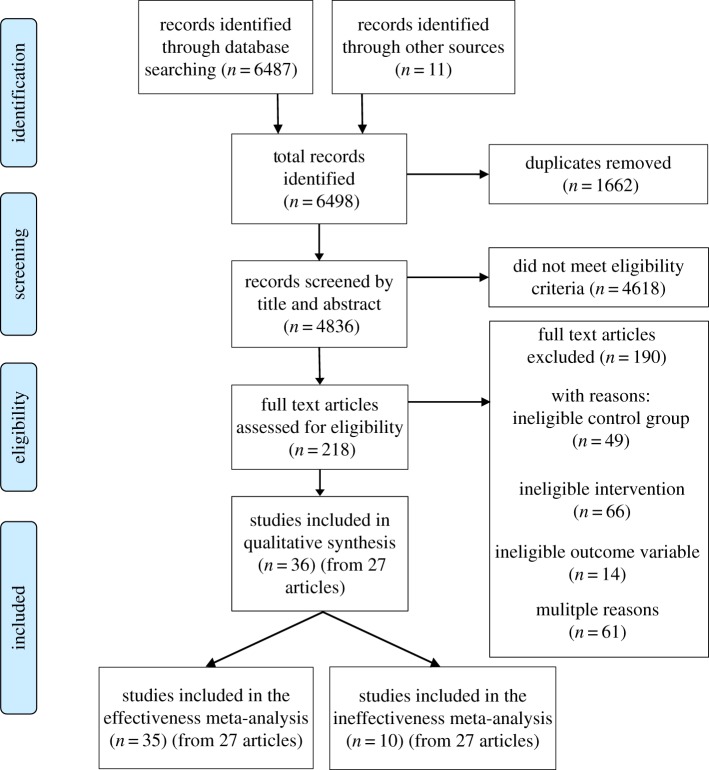
PRISMA flow chart displaying study flow.

Figure 1 displays the flow of studies through the systematic review process. A total of 4836 study records were screened based on their titles and abstracts. Full-text screening of 218 articles that were judged to be potentially eligible resulted in 26 that met the inclusion criteria. One further eligible article was identified after contact with authors, resulting in 27 articles. Within these 27 articles, 35 studies were eligible for the effectiveness meta-analysis (*N* = 30 858), 10 studies were eligible for the ineffectiveness meta-analysis (*N* = 5078), and one study—that communicated evidence of effectiveness—included insufficient information to be included in the meta-analysis [62].

### 3.2. Study characteristics

Of the 35 effect sizes included in the effectiveness meta-analysis, 20 were in the domain of health policies, nine in environmental policies, and six in other areas, including gun crime and education. Of the 10 effect sizes included in the ineffectiveness meta-analysis, six were in the domain of health policies, three in environmental policies, and one in another area—war. The majority of the studies were conducted in the USA (25/36) and despite the eligibility criteria being open to other designs, all included studies were randomized between-participants experiments. The control groups in most included studies (33/36) received no additional information beyond background information and an introduction to the policy(s). The remaining three studies provided information to control groups that the intervention groups did not receive, e.g. information about the planet Pluto. Further details on the characteristics of eligible studies are reported in electronic supplementary material, tables S1 and S2.

### 3.3. Risk of bias within studies

The majority of the studies were at high risk of bias. For the evidence of effectiveness meta-analysis, 26 studies were rated as weak (high risk of bias), nine were rated as moderate, and none were rated as strong (low risk of bias). The most common reason for this was a lack of validity and reliability testing for the main outcome variable (policy support). For the evidence of ineffectiveness meta-analysis, seven studies were rated as weak and three were rated as moderate.

### 3.4. Risk of bias across studies

**Figure 2.**
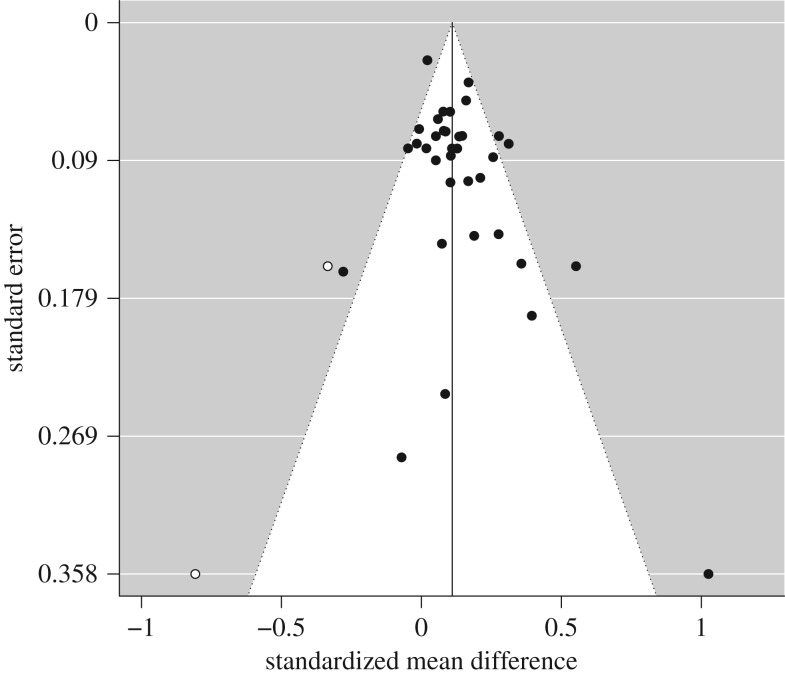
A funnel plot for the effectiveness meta-analysis with trim and fill.

Examination of the evidence of effectiveness funnel plots suggested possible asymmetry (figure 2). This was confirmed by Egger's regression, *Z* = 2.21, *p* = 0.027, that suggested a risk of bias across studies. The ‘trim and fill' technique was employed to account for this bias.

**Figure 3.**
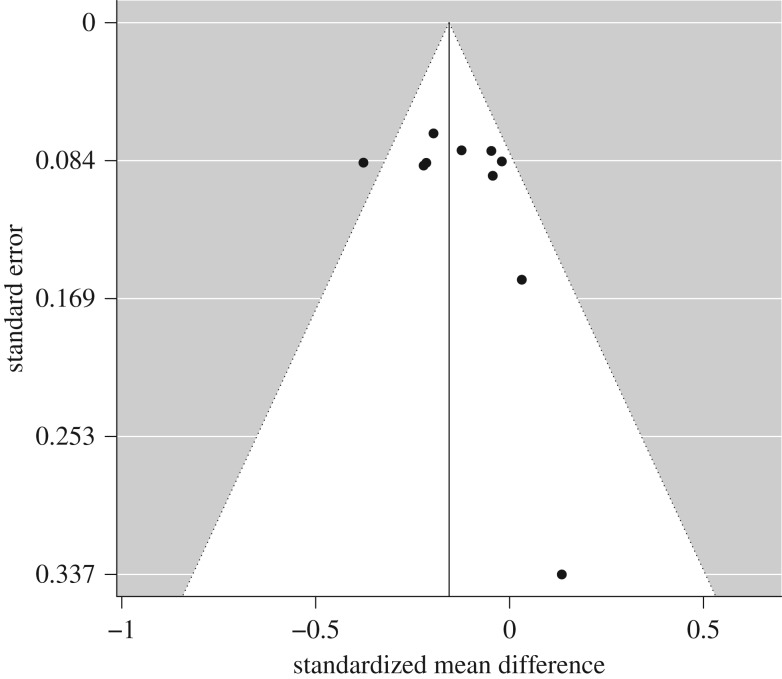
A funnel plot for the ineffectiveness meta-analysis without trim and fill.

Examination of the evidence of ineffectiveness funnel plots did not reveal any asymmetry (figure 3). This was confirmed by Egger's regression, *Z* = 1.22, *p* = 0.224, that suggested no risk of bias across studies.

### 3.5. Main results

#### 3.5.1. Communicating evidence of effectiveness

**Figure 4.**
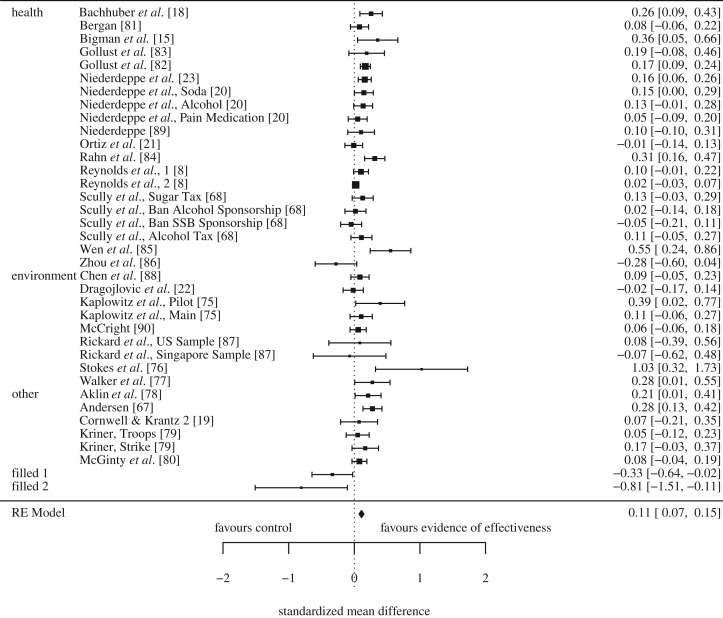
Forest plot of comparison: evidence of effectiveness communicated or no evidence of effectiveness communicated and support for the policy (corrected for bias using trim and fill).

Communicating evidence that a policy was effective in achieving a target outcome increased support for the policy, SMD = 0.11, 95% CI [0.07, 0.15], *p* < 0.0001 (figure 4). Assuming a normal distribution, this is equivalent to increasing support for a policy from 50% to 54% (95% CI [53%, 56%]; the change varies with levels of baseline support as shown in table 2). There was moderate and significant heterogeneity, *Q* (36) = 85.51, *p* < 0.001, *I^2^* = 47%, *T* = 0.071, *T^2^* = 0.005. This suggests that the intervention effects vary more than would be expected by chance alone. Due to an asymmetrical funnel plot, the trim and fill method was used. This did not substantively change the results, SMD = 0.12, 95% CI [0.08, 0.15], *p* < 0.0001 (without trim and fill).

##### 3.5.1.1. Sensitivity analysis

The main analysis was re-run to test whether the significant overall effect remained after (i) excluding the studies that were at high risk of bias, and (ii) excluding studies that contained confounded interventions. Excluding studies at high risk of bias resulted in *k* = 9 effect sizes and *N* = 12 527. Communicating evidence that a policy was effective increased support for the policy, SMD = 0.12, 95% CI [0.04, 0.20], *p* = 0.002. There was substantial and significant heterogeneity, *Q* (8) = 27.06, *p* < 0.001, *I^2^* = 65%, *T* = 0.09, *T^2^* = 0.01.

Excluding studies that had confounded interventions resulted in *k* = 11 effect sizes and *N* = 5870. There was no funnel plot asymmetry and therefore trim and fill was not used. The effect of communicating evidence of effectiveness increased support for the policies among these studies, SMD = 0.12, 95% CI [0.04, 0.19], *p* = 0.002. There was no significant heterogeneity among these studies, *Q* (10) = 15.58, *p* = 0.112, *I^2^* = 37%, *T* = 0.07, *T^2^* = 0.005.

##### 3.5.1.2. Moderator analyses

As seen in table 1, there was no evidence that policy domain, presentation of effectiveness, readability or the presence of uncertainty moderated the size of the effects.

**Table 1.** Meta-regressions of the moderation analysis on the effect communicating effectiveness and policy support.

	*B*	s.e.	95% CI	*p*-value
policy domain (reference = health)^a^				
environment	−0.04	0.06	[−0.15, 0.07]	0.478
other	0.01	0.05	[−0.09, 0.12]	0.115
presentation of effectiveness	−0.01	0.04	[−0.09, 0.07]	0.725
readability	0.01	0.01	[−0.01, 0.02]	0.311
presence of uncertainty^a^	0.04	0.04	[−0.05, 0.12]	0.394

^a^Study variance included in the regression due to heterogeneity.

#### 3.5.2. Communicating evidence of ineffectiveness

**Figure 5.**
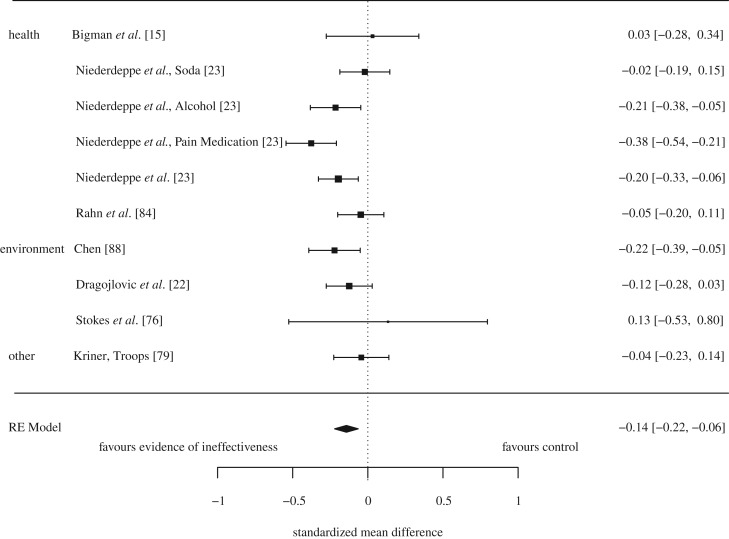
Forest plot of comparison: evidence of ineffectiveness communicated or no evidence of ineffectiveness communicated and support for the policy.

Communicating evidence that a policy was ineffective or leads to undesirable outcomes decreased support for the policy, SMD = −0.14, 95% CI [−0.22, −0.06], *p* < 0.001 (figure 5). Assuming a normal distribution, this is equivalent to support for a policy decreasing from 50% to 44% (95% CI [41%, 47%]; the change varies with levels of baseline support as shown in table 2). There was moderate and marginally significant heterogeneity, *Q* (9) = 16.27, *p =* 0.062, *I^2^* = 46%, *T* = 0.08, *T^2^* = 0.007. This suggests that the intervention effects vary marginally more so than would be expected by chance alone. There were insufficient studies to run moderation and sensitivity-by-covariate meta-analyses.

**Table 2.** Estimated change in support (% [95% CI]) depending on (i) initial support for the policy and (ii) communication of evidence of effectiveness or ineffectiveness.

initial support for the policy	support for the policy after communicating effectiveness	support for the policy after communicating ineffectiveness
10	12 (11, 13)	8 (7, 9)
20	23 (22, 24)	16 (14, 18)
30	34 (33, 35)	25 (23, 28)
40	44 (43, 46)	35 (32, 38)
50	54 (53, 56)	44 (41, 47)
60	64 (63, 66)	54 (51, 58)
70	74 (73, 75)	65 (62, 68)
80	83 (82, 84)	76 (73, 78)
90	92 (91, 92)	87 (86, 89)

##### 3.5.2.1. Sensitivity analysis

The main analysis was re-run to test whether the significant overall effect remained after excluding the studies that were at high risk of bias. Excluding studies at high risk of bias resulted in *k* = 3 effect sizes and *N* = 1198. There was no evidence that communicating evidence of ineffectiveness on policy changed support for policies, SMD = −0.08, 95% CI [−0.20, 0.03], *p* = 0.155. There was no evidence of heterogeneity, *Q* (2) = 0.87, *p* = 0.648, *I^2^* = 0%, *T* = 0.00, *T^2^* = 0.00.

## 4. Discussion

The results of this systematic review show that public support for a policy can be increased by communicating evidence of its effectiveness. Policies relating to health, environment and gun crime were examined, among others, and the results appear to be robust across the different domains. The results also do not significantly vary with intervention characteristics, including whether evidence of effectiveness was presented alone or confounded with other information, whether uncertainty was expressed when communicating evidence, the readability of the intervention, or whether the statement of effectiveness was asserted or described (quantitatively or qualitatively). These results also suggest that public support can be decreased by communicating evidence that the policy is ineffective or has undesirable outcomes, such as costing money to implement. Due to the small number of studies in this second meta-analysis, it was not possible to test whether the effect varies based on study and intervention characteristics. In contrast to our predictions that communicating ineffectiveness would lead to greater changes, the effect sizes estimated by these two meta-analyses were similar. With a policy that receives approximately 50% support—such as a levy on sugar-sweetened beverages [13]—communicating (in)effectiveness could increase support to 54% or decrease support to 44%.

Although the effect-size may seem relatively modest, small effects can have large real-world consequences [63]. To contextualize this, consider the role of evidence in driving public acceptability of major policy proposals, such as the UK European Union (EU) membership referendum held in 2016, where differences in public support came down to 3.8% (51.9% versus 48.1%) in favour of leaving the EU. In referenda, there is a pre-specified threshold of public support at which the government decides to implement the particular course of action. With other policy matters the threshold is not clear. While there is evidence demonstrating the importance of public support for enabling the implementation of a policy [4–6,64,65], there is uncertainty regarding the amount of support that is needed, with differences across countries to be expected. Despite this uncertainty, the current study does suggest that communicating evidence of policy effectiveness can form one part of an advocacy strategy designed to enable the implementation of effective policies. By contrast, communicating evidence of ineffectiveness appears to be able to reduce support to a similar degree. Real-world examples of this are plentiful, with a number of media outlets around the world reporting stories that gun control does not cut gun crime, the sugar tax does not reduce obesity, or that solar power is inefficient and unreliable. These messages are likely to damage support for these policies.

The changes that were found in public support in the current study were probably driven by a change in beliefs about a policy's effectiveness. Only one study in this review examined this directly: perceived effectiveness was the mechanism behind the change in support [8]. Across three studies, Reynolds *et al*. communicated evidence that a tax on confectionery would impact childhood obesity and/or inequalities in childhood obesity. Although not all of the interventions had the desired impact on public support, the results suggest that when public support was increased, it was primarily mediated by a change in the belief that the tax was effective at reducing childhood obesity. One further study also demonstrated that communicating evidence of the policy's effectiveness increased both perceptions of policy effectiveness and support for the policy, consistent with the mechanism interpretation [15].

These results also touch on the question about whether people update their beliefs and attitudes when exposed to evidence that contradicts or supports their pre-existing beliefs. There have been concerns that when the people are given evidence, they either ignore it or become even more entrenched in their current beliefs (i.e. a backfire effect; [27–29]). Contrary to this, the current findings support recent work suggesting that people's beliefs and attitudes are somewhat amenable to change [31–33,37,38,66]. However, using the current methods, it is not possible to determine which of the participants changed their beliefs and attitudes. The results of the current study could be interpreted as showing that on average exposing people to evidence can shift their views to reflect the evidence.

### 4.1. Strengths and limitations

The current study is the first of its kind to synthesize the available evidence concerning the impact of communicating evidence of a policy's effectiveness on public support. Given the mixed results of interventions that contain evidence of effectiveness [18–20,67], the current study provides the strongest evidence yet that communicating evidence that a policy is effective can increase support for a range of different policies.

While conducting this review at least two of the authors made decisions about study inclusion, data extraction, coding and quality assessment. When the decision was ambiguous or the two authors disagreed, a third, fourth, or occasionally a fifth reviewer would enter the discussion to resolve the decision. This process minimizes the likelihood of errors that arises from single reviewer decisions [58].

The majority of the studies in the effectiveness meta-analysis (26/35) and ineffectiveness meta-analysis (7/10) were at high risk of bias. Interpretations of the pooled estimates should therefore be treated cautiously. However, despite this overall rating, there is reason to believe that this may be a harsh description of the included studies. The included studies were all randomized experiments and many included a nationally representative sample. The quality assessment tool assesses a range of different factors. In particular, one of these factors received lower quality ratings across studies: outcome validity and reliability. Only one study reported tests that their outcome measure was valid [67]. Despite the outcome measures across studies being largely similar, the validity test of this one study could not be used to validate the measures used in the other studies. Future research would benefit from improving reporting standards, including confirming the validity and reliability of policy support measures.

There was moderate heterogeneity in the primary meta-analysis and this needs to be considered when interpreting the findings. A proportion of this heterogeneity was due to interventions that include additional information (other than evidence of effectiveness) such as information about the magnitude or consequences of the problem that is being targeted. In a sensitivity analysis, interventions that only included evidence of effectiveness were analysed, and there was no significant heterogeneity. This suggests that the effect sizes are consistent when communicating messages solely about policy effectiveness.

There was some evidence of funnel plot asymmetry for the evidence of effectiveness meta-analysis but not for the evidence of ineffectiveness meta-analysis. This may indicate publication bias, but other sources of bias could also explain this, such as language bias and outcome reporting bias [58]. We addressed this by using the ‘trim and fill' approach. While this does not eradicate the problem it can reduce it.

The majority of the primary research included in this review was conducted in the USA (25/36) and the majority of policies studied were in the domain of health (20/36). The results of the review may therefore be less generalizable to communicating evidence of effectiveness for policies other than health in countries other than the USA. However, in the meta-regression we did not find evidence that policy domain moderated the effect of communicating evidence but we note that this analysis had too low power to fully exclude this possibility.

### 4.2. Future research

Current studies preclude an estimate of effects in real-world settings, where multiple and competing messages are likely to be present. If a government desired to implement policies then competing messages may originate from a variety of sources. The results of the ineffectiveness meta-analysis suggest that opponents of policy can find success in communicating evidence that a policy is ineffective, which leads to reductions in support that are comparable in magnitude to communicating evidence of effectiveness. Online experiments have attempted to simulate how people respond to competing messages [20,68,69], and while this is a step forward in examining how competing messages are interpreted, this does not replicate how information is processed in the real world. Building on the work of Niederdeppe *et al*. would be a useful direction for this goal.

The meta-regressions conducted here suggested that the effect of communicating evidence on public support was not moderated by several intervention characteristics. However, this was based on associations rather than experimentation, and with a relatively small number of included studies, and a large number of confounding variables. Further research should investigate optimal communication methods from the perspectives of comprehension, accuracy and successful belief change [8,48]. One aspect of optimal communication may involve audience segmentation or targeted communication [70]. Personalized risk communication for changing behaviour has been shown to be either ineffective or of little effectiveness [71,72]. Nonetheless, psychographic targeting may have potential [73]. This approach suggests that tailoring a communication to the recipient's personality may increase the magnitude of any change. Currently this approach lacks a solid evidence base. Additionally it has recently come under ethical scrutiny [74]. More acceptable approaches would be those that enable recipients to make more informed decisions rather than to simply persuade them one way or another.

## 5. Conclusion

In summary, this review provides the most robust evidence to date that communicating evidence that a policy is effective can increase support for the policy, and comparable decreases in support can be achieved by communicating evidence that the policy is not effective. These changes in support may typically be considered as small, yet could be meaningful when considered at the population level. Presidential elections and referenda decisions have hung on less.

## Supplementary Material

Supplement Tables

Reviewer comments

## Data accessibility

The data and code are available at https://osf.io/4gjur/.

## References

[1] Wang YC, McPherson K, Marsh T, Gortmaker SL, Brown M 2011 Health and economic burden of the projected obesity trends in the USA and the UK. Lancet 378, 815–825. (10.1016/s0140-6736(11)60814-3) 21872750

[2] IPCC. 2018 *Global warming of 1.5°C*. An IPCC Special Report on the impacts of global warming of 1.5°C above pre-industrial levels and related global greenhouse gas emission pathways, in the context of strengthening the global response to the threat of climate change, sustainable development, and efforts to eradicate poverty. Geneva, Switzerland: IPCC.

[3] Gun Violence Archive. 2017 Mass shootings in 2017. See https://www.gunviolencearchive.org/reports/mass-shooting?year=2017.

[4] Cairney P 2009 The role of ideas in policy transfer: the case of UK smoking bans since devolution. J. Eur. Public Policy 16, 471–488. (10.1080/13501760802684718)

[5] Cullerton K, Donnet T, Lee A, Gallegos D 2016 Playing the policy game: a review of the barriers to and enablers of nutrition policy change. Public Health Nutr. 19, 2643–2653. (10.1017/S1368980016000677) 27034196 PMC10271040

[6] Cullerton K, Donnet T, Lee A, Gallegos D 2018 Effective advocacy strategies for influencing government nutrition policy: a conceptual model. Int. J. Behav. Nutr. Phys. Activity 15, 83 (10.1186/s12966-018-0716-y) PMC6119246 30170610

[7] Druckman JN, McGrath MC 2019 The evidence for motivated reasoning in climate change preference formation. Nat. Clim. Change 9, 111–119. (10.1038/s41558-018-0360-1)

[8] Reynolds JP, Pilling M, Marteau TM 2018 Communicating quantitative evidence of policy effectiveness and support for the policy: three experimental studies. Social Sci. Med. 218, 1–12. (10.1016/j.socscimed.2018.09.037) 30312911

[9] Bos C, Lans IV, Van Rijnsoever F, Van Trijp H 2015 Consumer acceptance of population-level intervention strategies for healthy food choices: the role of perceived effectiveness and perceived fairness. Nutrients 7, 7842–7862. (10.3390/nu7095370) 26389949 PMC4586565

[10] Eriksson L, Garvill J, Nordlund AM 2008 Acceptability of single and combined transport policy measures: the importance of environmental and policy specific beliefs. Transp. Res. Part A: Policy Pract. 42, 1117–1128. (10.1016/j.tra.2008.03.006)

[11] Lam S-P 2014 Predicting support of climate policies by using a protection motivation model. Clim. Policy 15, 321–338. (10.1080/14693062.2014.916599)

[12] Mazzocchi M, Cagnone S, Bech-Larsen T, Niedzwiedzka B, Saba A, Shankar B, Verbeke W, Traill WB 2015 What is the public appetite for healthy eating policies? Evidence from a cross-European survey. Health Econ. Policy Law 10, 267–292. (10.1017/S1744133114000346) 25170630

[13] Petrescu DC, Hollands GJ, Couturier DL, Ng YL, Marteau TM 2016 Public acceptability in the UK and USA of nudging to reduce obesity: the example of reducing sugar-sweetened beverages consumption. PLoS ONE 11, e0155995 (10.1371/journal.pone.0155995) 27276222 PMC4898693

[14] Storvoll EE, Moan IS, Rise J 2015 Predicting attitudes toward a restrictive alcohol policy: using a model of distal and proximal predictors. Psychol. Addictive Behav. 29, 492–499. (10.1037/adb0000036) 25347014

[15] Bigman CA, Cappella JN, Hornik RC 2010 Effective or ineffective: attribute framing and the human papillomavirus (HPV) vaccine. Patient Educ. Couns. 81, S70–S76. (10.1016/j.pec.2010.08.014) 20851560 PMC2993779

[16] Pechey R, Burge P, Mentzakis E, Suhrcke M, Marteau TM 2014 Public acceptability of population-level interventions to reduce alcohol consumption: a discrete choice experiment. Social Sci. Med. 113, 104–109. (10.1016/j.socscimed.2014.05.010) PMC4065329 24858928

[17] Promberger M, Dolan P, Marteau TM 2012 Pay them if it works: discrete choice experiments on the acceptability of financial incentives to change health related behaviour. Social Sci. Med. 75, 2509–2514. (10.1016/j.socscimed.2012.09.033) PMC3686527 23102753

[18] Bachhuber MA, McGinty EE, Kennedy-Hendricks A, Niederdeppe J, Barry CL 2015 Messaging to increase public support for naloxone distribution policies in the United States: results from a randomized survey experiment. PLoS ONE 10, e0130050 (10.1371/journal.pone.0130050) 26132859 PMC4488484

[19] Cornwell JF, Krantz DH 2014 Public policy for thee, but not for me: varying the grammatical person of public policy justifications influences their support. Judgment Deci. Making 9, 433.

[20] Niederdeppe J, Heley K, Barry CL 2015 Inoculation and narrative strategies in competitive framing of three health policy issues. J. Commun. 65, 838–862. (10.1111/jcom.12162)

[21] Ortiz SE, Zimmerman FJ, Adler GJ Jr 2016 Increasing public support for food-industry related, obesity prevention policies: the role of a taste-engineering frame and contextualized values. Social Sci. Med. 156, 142–153. (10.1016/j.socscimed.2016.02.042) 27038322

[22] Dragojlovic N, Einsiedel E 2015 What drives public acceptance of second-generation biofuels? Evidence from Canada. Biomass Bioenergy 75, 201–212. (10.1016/j.biombioe.2015.02.020)

[23] Niederdeppe J, Shapiro MA, Kim HK, Bartolo D, Porticella N 2014 Narrative persuasion, causality, complex integration, and support for obesity policy. Health Commun. 29, 431–444. (10.1080/10410236.2012.761805) 23829316

[24] Fishbein M, Ajzen I 1977 Belief, attitude, intention, and behavior: An introduction to theory and research.

[25] Lewandowsky S, Ecker UK, Cook J 2017 Beyond misinformation: understanding and coping with the ‘post-truth’ era. J. Appl. Res. Mem. Cognit. 6, 353–369. (10.1016/j.jarmac.2017.07.008)

[26] Kunda Z 1990 The case for motivated reasoning. Psychol. Bull. 108, 480 (10.1037/0033-2909.108.3.480) 2270237

[27] Nickerson RS 1998 Confirmation bias: a ubiquitous phenomenon in many guises. Rev. General Psychol. 2, 175–220. (10.1037/1089-2680.2.2.175)

[28] Lord CG, Ross L, Lepper MR 1979 Biased assimilation and attitude polarization: the effects of prior theories on subsequently considered evidence. J. Pers. Soc. Psychol. 37, 2098 (10.1037/0022-3514.37.11.2098)

[29] Nyhan B, Reifler J 2010 When corrections fail: the persistence of political misperceptions. Political Behav. 32, 303–330. (10.1007/s11109-010-9112-2)

[30] Kuhn D, Lao J 1996 Effects of evidence on attitudes: is polarization the norm? Psychol. Sci. 7, 115–120. (10.1111/j.1467-9280.1996.tb00340.x)

[31] van der Linden S, Leiserowitz A, Maibach E 2018 Scientific agreement can neutralize politicization of facts. Nat. Hum. Behav. 2, 2 (10.1038/s41562-017-0259-2) 30980051

[32] Wood T, Porter E 2018 The elusive backfire effect: mass attitudes' steadfast factual adherence. Political Behav. 41, 135–163. (10.1007/s11109-018-9443-y)

[33] Chan MS, Jones CR, Hall Jamieson K, Albarracín D 2017 Debunking: a meta-analysis of the psychological efficacy of messages countering misinformation. Psychol. Sci. 28, 1531–1546. (10.1177/0956797617714579) 28895452 PMC5673564

[34] Porter E, Wood TJ, Kirby D 2018 Sex trafficking, Russian infiltration, birth certificates, and pedophilia: a survey experiment correcting fake news. J. Exp. Political Sci. 5, 159–164. (10.1017/XPS.2017.32)

[35] van der Linden SL, Leiserowitz AA, Feinberg GD, Maibach EW 2015 The scientific consensus on climate change as a gateway belief: experimental evidence. PLoS ONE 10, e0118489 (10.1371/journal.pone.0118489) 25714347 PMC4340922

[36] Nyhan B, Reifler J 2015 Does correcting myths about the flu vaccine work? An experimental evaluation of the effects of corrective information. Vaccine 33, 459–464. (10.1016/j.vaccine.2014.11.017) 25499651

[37] Kalinoski ZT, Steele-Johnson D, Peyton EJ, Leas KA, Steinke J, Bowling NA 2013 A meta-analytic evaluation of diversity training outcomes. J. Organ. Behav. 34, 1076–1104. (10.1002/job.1839)

[38] Steinmetz H, Knappstein M, Ajzen I, Schmidt P, Kabst R 2016 How effective are behavior change interventions based on the theory of planned behavior? Zeitschrift für Psychologie 224, 216–233. (10.1027/2151-2604/a000255)

[39] Vosoughi S, Roy D, Aral S 2018 The spread of true and false news online. Science 359, 1146–1151. (10.1126/science.aap9559) 29590045

[40] Del Vicario M, Vivaldo G, Bessi A, Zollo F, Scala A, Caldarelli G, Quattrociocchi W 2016 Echo chambers: emotional contagion and group polarization on Facebook. Sci. Rep. 6, 37825 (10.1038/srep37825) 27905402 PMC5131349

[41] Zollo F, Bessi A, Del Vicario M, Scala A, Caldarelli G, Shekhtman L, Havlin S, Quattrociocchi W 2017 Debunking in a world of tribes. PLoS ONE 12, e0181821 (10.1371/journal.pone.0181821) 28742163 PMC5524392

[42] Barberá P, Jost JT, Nagler J, Tucker JA, Bonneau R 2015 Tweeting from left to right: is online political communication more than an echo chamber? Psychol. Sci. 26, 1531–1542. (10.1177/0956797615594620) 26297377

[43] Nyhan B, Reifler J, Ubel PA 2013 The hazards of correcting myths about health care reform. Med. Care 51, 127–132. (10.1097/MLR.0b013e318279486b) 23211778

[44] Mason L 2018 Ideologues without issues: the polarizing consequences of ideological identities. Public Opinion Q. 82, 280–301. (10.1093/poq/nfy005)

[45] Chong D, Druckman JN 2007 Framing theory. Annu. Rev. Political Sci. 10, 103–126. (10.1146/annurev.polisci.10.072805.103054)

[46] Petty RE, Cacioppo JT 1986 The elaboration likelihood model of persuasion, pp. 123–205. Springer Series in Social Psychology. New York, NY: Springer.

[47] Berry DC, Hochhauser M 2006 Verbal labels can triple perceived risk in clinical trials. Drug Info. J. 40, 249–258. (10.1177/009286150604000302)

[48] Spiegelhalter D 2017 Risk and uncertainty communication. Annu. Rev. Stat. Appl. 4, 31–60. (10.1146/annurev-statistics-010814-020148)

[49] Løhre E, Teigen KH 2016 There is a 60% probability, but I am 70% certain: communicative consequences of external and internal expressions of uncertainty. Think. Reason. 22, 369–396. (10.1080/13546783.2015.1069758)

[50] Jenkins SC, Harris AJ, Lark R 2018 Understanding ‘unlikely (20% likelihood)’ or ‘20% likelihood (unlikely)’ outcomes: The robustness of the extremity effect. J. Behav. Decis. Mak. 31, 572–586. (10.1002/bdm.2072)

[51] Tversky A, Kahneman D 1974 Judgment under uncertainty: heuristics and biases. Science 185, 1124–1131. (10.1126/science.185.4157.1124) 17835457

[52] Rozin P, Royzman EB 2001 Negativity bias, negativity dominance, and contagion. Pers. Soc. Psychol. Rev. 5, 296–320. (10.1207/S15327957PSPR0504_2)

[53] Moher D, Liberati A, Tetzlaff J, Altman DG, the Prisma Group. 2009 Preferred reporting items for systematic reviews and meta-analyses: the PRISMA statement. PLoS Med. 6, e1000097 (10.1371/journal.pmed.1000097) 19621072 PMC2707599

[54] Ardanaz M, Murillo MV, Pinto PM 2013 Sensitivity to issue framing on trade policy preferences: evidence from a survey experiment. Int. Organ. 67, 411–437. (10.1017/S0020818313000076)

[55] Borenstein M, Hedges LV, Higgins JP, Rothstein HR 2011 Introduction to meta-analysis. West Sussex, UK: John Wiley & Sons.

[56] McCright AM, Charters M, Dentzman K, Dietz T 2016 Examining the effectiveness of climate change frames in the face of a climate change denial counter-frame. Top. Cogn. Sci. 8, 76–97. (10.1111/tops.12171) 26621098

[57] Effective Public Health Practice Project. 1998 Quality assessment tool for quantitative studies.

[58] Higgins JP, Green S 2008 Cochrane handbook for systematic reviews of interventions. West Sussex, UK: John Wiley & Sons.

[59] Ding T, Baio G 2018 bmeta: Bayesian Meta-Analysis and Meta-Regression. See https://cran.r-project.org/web/packages/bmeta/bmeta.pdf.

[60] Viechtbauer W 2010 Conducting meta-analyses in R with the metafor package. J. Stat. Softw. 36, 1–48. (10.18637/jss.v036.i03)

[61] Furukawa TA, Leucht S 2011 How to obtain NNT from Cohen's d: comparison of two methods. PLoS ONE 6, e19070 (10.1371/journal.pone.0019070) 21556361 PMC3083419

[62] Facchini G, Margalit Y, Nakata H. 2016 Countering public opposition to immigration: The impact of information campaigns. *SSRN*. See http://ftp.iza.org/dp10420.pdf.

[63] Prentice DA, Miller DT 1992 When small effects are impressive. Psychol. Bull. 112, 160 (10.1037/0033-2909.112.1.160)

[64] Roache SA, Gostin LO 2018 Tapping the power of soda taxes: a call for multidisciplinary research and broad-based advocacy coalitions—a response to the recent commentaries. Int. J. Health Policy Manage. 7, 674–676. (10.15171/ijhpm.2018.30) PMC6037498 29996590

[65] Smith K 2013 Beyond evidence based policy in public health: the interplay of ideas. Berlin, Germany: Springer.

[66] Albarracin D, Shavitt S 2018 Attitudes and attitude change. Annu. Rev. Psychol. 69, 299–327. (10.1146/annurev-psych-122216-011911) 28841390 PMC12479097

[67] Andersen SC 2017 From passive to active representation—experimental evidence on the role of normative values in shaping white and minority bureaucrats' policy attitudes. J. Public Adm. Res. Theory 27, 400–414. (10.1093/jopart/mux006)

[68] Scully M, Brennan E, Durkin S, Dixon H, Wakefield M, Barry CL, Niederdeppe J 2017 Competing with big business: a randomised experiment testing the effects of messages to promote alcohol and sugary drink control policy. BMC Public Health 17, 945 (10.1186/s12889-017-4972-6) 29282031 PMC5745776

[69] Van der Linden S, Leiserowitz A, Rosenthal S, Maibach E 2017 Inoculating the public against misinformation about climate change. Global Chall. 1, 1600008 (10.1002/gch2.201600008) PMC6607159 31565263

[70] Hine DW, Reser JP, Morrison M, Phillips WJ, Nunn P, Cooksey R 2014 Audience segmentation and climate change communication: conceptual and methodological considerations. Wiley Interdiscip. Rev. Clim. Change 5, 441–459. (10.1002/wcc.279)

[71] Hollands GJ, French DP, Griffin SJ, Prevost AT, Sutton S, King S, Marteau TM 2016 The impact of communicating genetic risks of disease on risk-reducing health behaviour: systematic review with meta-analysis. Br. Med. J. 352, i1102 (10.1136/bmj.i1102) 26979548 PMC4793156

[72] Noar SM, Benac CN, Harris MS 2007 Does tailoring matter? Meta-analytic review of tailored print health behavior change interventions. Psychol. Bull. 133, 673 (10.1037/0033-2909.133.4.673) 17592961

[73] Matz SC, Kosinski M, Nave G, Stillwell DJ 2017 Psychological targeting as an effective approach to digital mass persuasion. Proc. Natl Acad. Sci. USA 114, 12 714–12 719. (10.1073/pnas.1710966114) PMC5715760 29133409

[74] Ruggeri K, Yoon H, Kácha O, van der Linden S, Muennig P 2017 Policy and population behavior in the age of Big Data. Curr. Opin. Behav. Sci. 18, 1–6. (10.1016/j.cobeha.2017.05.010)

[75] Kaplowitz SA, McCright AM 2015 Effects of policy characteristics and justifications on acceptance of a gasoline tax increase. Energy Policy 87, 370–381. (10.1016/j.enpol.2015.08.037)

[76] Stokes LC, Warshaw C 2017 Renewable energy policy design and framing influence public support in the United States. Nat. Energy 2, 17107 (10.1038/nenergy.2017.107)

[77] Walker BJ, Wiersma B, Bailey E 2014 Community benefits, framing and the social acceptance of offshore wind farms: an experimental study in England. Energy Res. Social Sci. 3, 46–54. (10.1016/j.erss.2014.07.003)

[78] Aklin M, Bayer P, Harish S, Urpelainen J 2014 Information and energy policy preferences: a survey experiment on public opinion about electricity pricing reform in rural India. Econ. Gov. 15, 305–327. (10.1007/s10101-014-0146-5)

[79] Kriner DL, Shen FX 2016 Conscription, inequality, and partisan support for war. J. Confl. Resolution 60, 1419–1445. (10.1177/0022002715590877)

[80] McGinty EE, Webster DW, Barry CL 2013 Effects of news media messages about mass shootings on attitudes toward persons with serious mental illness and public support for gun control policies. Am. J. Psychiatry 170, 494–501. (10.1176/appi.ajp.2013.13010014) 23511486

[81] Bergan DE 2012 Partisan stereotypes and policy attitudes. J. Commun. 62, 1102–1120. (10.1111/j.1460-2466.2012.01676.x)

[82] Gollust SE, Barry CL, Niederdeppe J 2017 Partisan responses to public health messages: motivated reasoning and sugary drink taxes. J. Health Polit. Policy Law 42, 1005–1037. (10.1215/03616878-4193606) 28801464

[83] Gollust SE, Tang X, White JM, French SA, Runge CF, Rothman AJ 2016 Young adults’ responses to alternative messages describing a sugar-sweetened beverage price increase. Public Health Nutr. 20, 46–52. (10.1017/S1368980016001816) 27465661 PMC10261550

[84] Rahn WM, Gollust SE, Tang X 2017 Framing food policy: the case of raw milk. Policy Studies J. 45, 359–383. (10.1111/psj.12161)

[85] Wen X, Higgins ST, Xie C, Epstein LH 2015 Improving public acceptability of using financial incentives for smoking cessation during pregnancy: a randomized controlled experiment. Nicotine Tob. Res. 18, 913–918. (10.1093/ntr/ntv204) 26385928

[86] Zhou S, Niederdeppe J 2017 The promises and pitfalls of personalization in narratives to promote social change. Commun. Monogr. 84, 319–342. (10.1080/03637751.2016.1246348)

[87] Rickard LN, Yang ZJ, Schuldt JP 2016 Here and now, there and then: how ‘departure dates’ influence climate change engagement. Global Environ. Change 38, 97–107. (10.1016/j.gloenvcha.2016.03.003)

[88] Chen D, Cheng C-y, Urpelainen J 2016 Support for renewable energy in China: a survey experiment with internet users. J. Clean. Prod. 112, 3750–3758. (10.1016/j.jclepro.2015.08.109)

[89] Niederdeppe J, Roh S, Dreisbach C 2016 How narrative focus and a statistical map shape health policy support among state legislators. Health commun. 31, 242–255. (10.1080/10410236.2014.998913) 26086340

[90] McCright AM, Charters M, Dentzman K, Dietz T 2016 Examining the effectiveness of climate change frames in the face of a climate change denial counter-frame. Topics Cogn. Sci. 8, 76–97. (10.1111/tops.12171) 26621098

