# Supplementary material for: Communicating the effectiveness and ineffectiveness of government policies and their impact on public support: a systematic review with meta-analysis
Source: R Soc Open Sci. 2020 Jan 15;7(1):190522. doi: 10.1098/rsos.190522 (PMC7029938; doi:10.1098/rsos.190522)
Supplement: Supplement Tables [file rsos190522supp1.docx]

# Supplement for

# Communicating the effectiveness and ineffectiveness of government policies and its impact on public support: A systematic review with meta-analysis

Table 1. Characteristics of included studies (evidence of effectiveness)

| Study | Methods | Participants | Policy area | Policy | Intervention message | Control message | Summary of results |
| --- | --- | --- | --- | --- | --- | --- | --- |
| Aklin 2014 | *Design*: Between-participants experiment  *Setting*: Household survey | *N* = 593  *Country*: India  *Age*: unclear  *Female*: unclear | Energy | Electricity pricing reform | Policy would lead to a more reliable power source | No additional text | “we see that statistically significant differences in means across  groups are found only for our second outcome variable” |
| Andersen 2017 | *Design*: Between-participants experiment  *Setting*: Online survey | *N* = 1096  *Country*: USA  *Age*: unclear  *Female*: 59%  *Other:* School principles | Education | Instigate a multicultural curriculum | Policy would reduce children’s prejudices | No additional text | “support for the policy is enhanced when they are  presented with the information” |
| Bachhuber 2015 | *Design*: Between-participants experiment  *Setting*: Online survey | *N* = 1598  *Country*: USA  *Age*: 10% (18-25), 16% (25-34), 16% (35-44), 17% (45-54), 22% (55-64), 19% (65+)  *Female*: 52% | Health | Naloxone distribution | Policy would reduce drug overdose deaths | No additional text | “Factual information alone led to  higher support” |
| Bergan 2012 | *Design*: Between-participants experiment  *Setting*: Online survey | *N* = 1302  *Country*: USA  *Age*: unclear  *Female*: unclear | Health | Health insurance exchange | Policy would increase the number of people with insurance while lowering costs | No additional text | Comparison of interest not summarised |
| Bigman 2010 | *Design*: Between-participants experiment  *Setting*: Online survey | *N* = 334  *Country*: USA  *Age*: mean = 50 (SD = 16)  *Female*: 52% | Health | Mandate HPV vaccination | Policy would prevent cervical cancer | No additional text | “Those who read about vaccine effectiveness agreed more that the HPV vaccine was an effective way to prevent cervical cancer. They tended to have a more positive attitude toward use of the vaccination as a way to prevent cervical cancer.” |
| Chen 2016 | *Design*: Between-participants experiment  *Setting*: Online survey | *N* = 2086  *Country*: China  *Age*: mean = 31.36 (SD = 7.76)  *Female*: 51% | Energy / Environment | Invest in renewable energy | Policy would reduce pollution/ reduce greenhouse gas/ improve energy security | No additional text | “the energy security treatment is statistically distinguishable from the pure control. The  other treatment groups are far from having statistically significant effects compared to the pure control” |
| Cornwell & Krantz 2014 | *Design*: Between-participants experiment  *Setting*: Online survey | *N* = 296  *Country*: USA  *Age*: unclear  *Female*: unclear | Multiple | 16 different policies | Varied | No additional text | The third-person evidence increased support for policies. The second-person evidence was no better than giving no evidence at all. |
| Dragojlovic 2015 | *Design*: Between-participants experiment  *Setting*: Online survey | *N* = 1302  *Country*: Canada  *Age*: mean = 53.5  *Female*: 51.4% | Energy / Environment | Mandates / subsidies for biofuel | Policy would help reduce climate change | No additional text | “the climate change argument in favor of  biofuels did not influence policy support” |
| Facchini 2016 | *Design*: Between-participants experiment  *Setting*: Online survey | *N* = 6000  *Country*: Japan  *Age*: unclear  *Female*: unclear | Immigration | Increase immigration | Policy would increase the number of workers in the country / increase the number of care workers / increase population size / increase the number of workers / increase the number of IT engineers / allow Japan to have a similar immigration rate to other advanced nations | Information about the planet Pluto | “providing information on some of the  economic benefits of immigration has a large, positive and significant effect on individual  opinions” |
| Gollust 2016 | *Design*: Between-participants experiment  *Setting*: Online survey | *N* = 494  *Country*: USA  *Age*: 21% (18), 24% (19), 20% (20), 23% (21-22), 9.3% (23-30), 2% (30+)  *Female*: 69.4%  *Transgender*: 0.2% | Health | Sugary drink tax | Policy would Reduce obesity/ Reduce budget deficit/ Fund oral health costs/ Fund chronic health care costs/ Protect children | No additional text | “No significant differences by message were observed for  the soda tax policy support measure” |
| Gollust 2017 | *Design*: Between-participants experiment  *Setting*: Online survey | *N* = 5147  *Country*: USA  *Age*: unclear  *Female*: unclear | Health | Sugary drink tax | Policy would raise money that could be used to combat obesity. | No additional text | “The protax  message showed no effects on tax support” |
| Kaplowitz 2015; Study 1 | *Design*: Between-participants experiment  *Setting*: Online survey | *N* = 237  *Country*: USA  *Age*: unclear  *Female*: unclear | Transport, Environment | $1 per gallon gasoline tax increase | Policy would reduce consumption/ raise money/ raise money to invest in energy efficient vehicles | No additional text | “Such support was positively influenced by (a) providing an argument that the tax increase will motivate the purchase of fuel efficient vehicles  Surprisingly, neither of the alternative uses of the extra revenue had statistically significant effects on support for the gasoline tax increase.” |
| Kaplowitz 2015; Study 2 | *Design*: Between-participants experiment  *Setting*: Online survey | *N* = 1068  *Country*: USA  *Age*: unclear  *Female*: unclear | Transport, Environment | $1 per gallon gasoline tax increase | Policy would reduce consumption/ raise money/ raise money to invest in energy efficient vehicles | No additional text | *See above. Summary of results are combined with Study 1* |
| Kriner 2015 (Study 1) | *Design*: Between-participants experiment  *Setting*: Online survey | *N* = 1200  *Country*: USA  *Age*: unclear  *Female*: unclear | Defence | Sending troops to South Korea | Policy would reduce inequalities in war casualties | No additional text | “Among this group, war support was slightly higher than in the simple draft treatment, However this difference in means fails to meet conventional levels of statistical significance” |
| Kriner 2015 (Study 2) | *Design*: Between-participants experiment  *Setting*: Online survey | *N* = 566  *Country*: USA  *Age*: unclear  *Female*: unclear | Defence | Going to war | Policy would reduce inequalities in war casualties | No additional text | “Support for the use of force was slightly higher, 37 percent, among those told that reinstituting the draft would lessen socioeconomic inequality in military sacrifice.” |
| McCright 2015 | *Design*: Between-participants experiment  *Setting*: Online survey | *N* = 1591  *Country*: USA  *Age*: unclear  *Female*: 45.9% | Environment | Reduce greenhouse gas emissions | Policy would benefit the economy/ national security/ God’s creation/ public health | No additional text | “Respondents exposed  to the economic opportunity frame do report greater support for aggressively attempting  to reduce our nation’s GHG emissions in the near future than do respondents not exposed to this positive frame—additional evidence that speaks to the potential of an economic opportunity frame in shifting Americans’ ACC views. However, in no other model does exposure to a positive frame have a statistically significant positive effect on any of the  ACC views. In other words, the overall potential of these positive frames for influencing  Americans’ ACC views is limited at best” |
| McGinty 2013 | *Design*: Between-participants experiment  *Setting*: Online survey | *N* = 1797  *Country*: USA  *Age*: 19% (18-29), 25% (30-44), 29% (45-59), 27% (60+)  *Female*: 51.4% | Crime | Gun restrictions for mental illness and ban on large capacity magazines | Policy would protect the public from gun crime | No additional text | “News media portrayals of mass shooting events by  persons with serious mental illness appear to play a critical  role in influencing both negative attitudes toward persons with serious mental illness and support for gun control policies. The same story raised public support for both gun restrictions for persons with serious mental illness and a ban on large capacity magazines” |
| Niederdeppe 2014 | *Design*: Between-participants experiment  *Setting*: Online survey | *N* = 3118  *Country*: USA  *Age*: 14% (18-24), 20% (25-34), 23% (35-44), 20% (45-54), 24% (55-64)  *Female*: 50.6% | Health | Sugary drinks tax | Policy would reduce sugary drink consumption and help reduce obesity | No additional text | “respondents exposed to the inoculation message  had significantly higher policy support than those in the no-exposure control group” |
| Niederdeppe 2015 | *Design*: Between-participants experiment  *Setting*: Online survey | *N* = 5007  *Country*: USA  *Age*: 16% (18-24), 24% (25-34), 16% (35-44), 14% (45-54), 18% (55-64), 13% (65+)  *Female*: 63.4% | Health | Warning labels | Policy would reduce cigarette use / reduce sugary drink consumption/ reduce abuse of pain medication | No additional text | “Those exposed to the inoculation message  and the narrative message had  greater policy support at t1 than those exposed to the control message” |
| Niederdeppe 2016 | *Design*: Between-participants experiment  *Setting*: Online survey | *N* = 496  *Country*: USA  *Age*: mean = 55.7 (SD = 11.7)  *Female*: 34.5% | Health | Incentives to encourage farmers markets, grants to encourage stores to sell healthy products, incentives for stores to open in communities with limited access to healthy foods | Policy would improve access to healthy foods and boost the local economy | No additional text | “None of the ANOVAs including all five conditions  found any statistically significant differences between any of  the four message conditions or and the no-exposure control  group” |
| Ortiz 2016 | *Design*: Between-participants experiment  *Setting*: Online survey | *N* = 2580  *Country*: USA  *Age*: 24% (18-29), 25% (30-44), 31% (45-60), 21% (60+)  *Female*: 54.5% | Health | Increasing healthy food availability, food advertising regulations, food labelling regulations | Policy would strengthen consumers' knowledge about food and drinks and enable them to make healthier decisions | No additional text | “No statistically significant treatment effects were found for any of the food and beverage policies.” |
| Rahn 2017 | *Design*: Between-participants experiment  *Setting*: Online survey | *N* = 1630  *Country*: USA  *Age*: 16% (18-29), 15% (30-39), 15% (40-49) 22% (50-59), 20% (60-69), 12% (70+)  *Female*: 66.1% | Health | Permit the sale of raw milk | Policy would enable consumers to have a milk that tastes better and offers health benefits | No additional text | “The difference between the control and the one-sided public health framing condition was not statistically significant… the level of mean support in the one-sided consumer choice treatment was significantly higher than in the control condition” |
| Reynolds 2018 Study 1 | *Design*: Between-participants experiment  *Setting*: Online survey | *N* = 1568  *Country*: England  *Age*: 16% (18-24), 18% (25-34), 19% (35-44), 19% (45-54), 28% (55+)  *Female*: 50.4% | Health | Tax on sweets and chocolates | Policy would reduce childhood obesity and inequalities in childhood obesity | No additional text | “None of the interventions affected support for the sweet tax.” |
| Reynolds 2018 Study 2 | *Design*: Between-participants experiment  *Setting*: Online survey | *N* = 7489  *Country*: UK  *Age*: 11% (18-24), 42% (25-49), 25% (50-65), 21% (66+)  *Female*: 51.3% |  | Tax on sweets and chocolates | Policy would reduce childhood obesity | No additional text | “Support for the tax did not increase in the basic infographic group but did in the enhanced group when compared to the control.” |
| Rickard 2016 | *Design*: Between-participants experiment  *Setting*: Online survey | *N* = 183  *Country*: Singapore  *Age*: mean = 22.44 (SD = 1.65)  *Female*: 71.0%  *N* = 193  *Country*: USA  *Age*: mean = 19.55 (SD = 1.28)  *Female*: 50.3% | Environment | 12 policies relating to climate change prevention | Policy would help mitigate climate change | No additional text | “mean-level policy support varied widely across conditions, from a low of M = 5.89 (SD = 1.43) (NYC_2047 condition, U.S. sample) to a high of M = 7.38 (SD = 1.12) (NYC_2066 condition, Singapore sample).” |
| Scully 2017 | *Design*: Between-participants experiment  *Setting*: Online survey | *N* = 6000  *Country*: Australia  *Age*: 8% (18-24), 14% (25-34), 14% (35-44), 16% (45-54), 22% (55-64), 27% (65+)  *Female*: 59.2% | Health | Taxes on alcohol and sugary drinks, banning of sports sponsorship for alcohol and sugary drinks | Policy would promote safer drinking culture/ improve peoples diet and health | Background information on the problem (differed by policy area) | “There was limited evidence of immediate effects of the inoculation and narrative messages above and beyond a message about the size and seriousness of the health issue (i.e. the control message), with only the narrative condition leading to higher target policy support after initial exposure.” |
| Stokes 2017 | *Design*: Between-participants experiment  *Setting*: Online survey | *N* = 2500  *Country*: USA  *Age*: 53% (<44)  *Female*: 54% | Environment | Renewable energy portfolios | Policy would create jobs/ reduce pollution | No additional text | “Support for an RPS would increase substantially if it led to a large increase in jobs. if citizens understood an RPS  bill would reduce harmful air pollution such as mercury, it would increase public support by the same amount” |
| Walker 2014 | *Design*: Between-participants experiment  *Setting*: Postal survey | *N* = 311  *Country*: UK  *Age*: mean = 54.3, median = 55  *Female*: 60.5%  *Gender not disclosed:* 2.9% | Environment | Offshore windfarm | Policy would benefit local communities and reduce energy bills | No additional text | “Support for the development was greatest under the community benefit frame” |
| Wen 2015 | *Design*: Between-participants experiment  *Setting*: Online survey | *N* = 166  *Country*: USA  *Age*: mean = 32.4 (SD = 9.9)  *Female*: 54.2% | Health | Financial incentives for smoking cessation | Policy would help pregnant women quit smoking | Information on car safety | “After the intervention, 27.7% more of participants in the intervention group and only 4.8% more in the control group agreed that Paying pregnant women who smoke to quit smoking is a good idea” |
| Zhou 2017 | *Design*: Between-participants experiment  *Setting*: Online survey | *N* = 368  *Country*: USA  *Age*: mean = 34 (SD = 11.63)  *Female*: 42% | Health | Incentives to encourage farmers markets, grants to encourage stores to sell healthy products, incentives for stores to open in communities with limited access to healthy foods | Policy would improve access to healthy foods and boost the local economy | No additional text | “The depersonalized story with unidentifiable collective characters and without inner states depictions produced higher levels of support for policies addressing food deserts than the no-exposure control group.” |

Table 2. Characteristics of included studies (evidence of ineffectiveness)

| Study | Methods | Participants | Policy area | Policy | Intervention message | Control message | Summary of results |
| --- | --- | --- | --- | --- | --- | --- | --- |
| Bigman 2010 | *Design*: Between-participants experiment  *Setting*: Online survey | *N* = 334  *Country*: USA  *Age*: mean = 50 (SD = 16)  *Female*: 52% | Health | Mandate HPV vaccination | Policy would be ineffective at preventing cervical cancer | No additional text | Comparison of interest not summarised |
| Chen 2016 | *Design*: Between-participants experiment  *Setting*: Online survey | *N* = 2086  *Country*: China  *Age*: mean = 31.36 (SD = 7.76)  *Female*: 51% | Energy / Environment | Invest in renewable energy | Policy would be too expensive | No additional text | “the economic counter-frame had a significant negative effect on support” |
| Dragojlovic 2015 | *Design*: Between-participants experiment  *Setting*: Online survey | *N* = 1302  *Country*: Canada  *Age*: mean = 53.5  *Female*: 51.4% | Energy / Environment | Mandates / subsidies for biofuel | Policy would increase the cost of food crops | No additional text | “We find that support for biofuels policies was  reduced in our experiment when respondents were exposed to an argument about the potential impact of biofuels production on food prices” |
| Kriner 2015 (Study 1) | *Design*: Between-participants experiment  *Setting*: Online survey | *N* = 1200  *Country*: USA  *Age*: unclear  *Female*: unclear | Defence | Sending troops to South Korea | Policy would be ineffective at reducing inequalities in war casualties | No additional text | “Support for the use of force was at its lowest ebb among respondents told that the draft would be used and that it would fail to reduce inequality in sacrifice; however, this figure, 40 percent, is not significantly different from the other draft treatments” |
| Niederdeppe 2014 | *Design*: Between-participants experiment  *Setting*: Online survey | *N* = 3118  *Country*: USA  *Age*: 14% (18-24), 20% (25-34), 23% (35-44), 20% (45-54), 24% (55-64)  *Female*: 50.6% | Health | Sugary drinks tax | Policy would be ineffective at reducing obesity | No additional text | “Tax support trended toward decline in almost all  conditions that featured a strong anti-tax frame” |
| Niederdeppe 2015 | *Design*: Between-participants experiment  *Setting*: Online survey | *N* = 5007  *Country*: USA  *Age*: 16% (18-24), 24% (25-34), 16% (35-44), 14% (45-54), 18% (55-64), 13% (65+)  *Female*: 63.4% | Health | Warning labels | Policy would be ineffective at reducing cigarette use / reducing sugary drink consumption/ reducing abuse of pain medication | No additional text | “respondents exposed to the anti-policy industry message at t1 had lower policy  support than those not exposed it at t1” |
| Rahn 2017 | *Design*: Between-participants experiment  *Setting*: Online survey | *N* = 1630  *Country*: USA  *Age*: 16% (18-29), 15% (30-39), 15% (40-49) 22% (50-59), 20% (60-69), 12% (70+)  *Female*: 66.1% | Health | Permit the sale of raw milk | Policy is not safe and would lead to illness | No additional text | “arguments about health  risks do not appear influential with the public” |
| Stokes 2017 | *Design*: Between-participants experiment  *Setting*: Online survey | *N* = 2500  *Country*: USA  *Age*: 53% (<44)  *Female*: 54% | Environment | Renewable energy portfolios | Policy would be ineffective at creating new jobs / would cost money | No additional text | “If, however, costs are imposed on residential ratepayers through rapid utility bill increases tied to renewable energy policies, this will very likely undermine support”  “We found that public support would drop significantly if an RPS did not create any jobs.” |

Table 3. Definitions and examples of different forms of communicating the effectiveness of policies

|  | Quantitative estimate | Qualitative estimate | Assertion |
| --- | --- | --- | --- |
| Definition | Provides numbers to describe the magnitude of the impact of the policy on a salient outcome | Provides words to describe the magnitude of the impact of the policy on a salient outcome | States that the policy has an impact on a salient outcome, but does not use numbers or words to describe the magnitude of this impact |
| *Studies* |  |  |  |
| Aklin 2014 |  | “According to these experts, increasing the price of electricity could provide Uttar Pradesh villages with a **more** reliable supply of power” |  |
| McCright 2015 | “… create **millions** of full-time blue-collar jobs that cannot be exported.” | “… **substantially** decrease our greenhouse gas emissions. Doing this will **significantly** increase our economic opportunities.” | “ Economic leaders argue that aggressively promoting energy efficiency and renewable energy technology will create new markets that will drive a new wave of innovation and entrepreneurialism” |
| Reynolds 2018 | “**0.8 of every 100** children are predicted to no longer be obese” |  |  |
| McGinty 2013 |  |  | "Getting this law in place is one way to protect the public from dangerous guns." |
| Niederdeppe 2015 |  |  | “Prohibiting stores located near schools from selling tobacco, requiring tobacco manufacturers to place large, graphic warning labels on the front of cigarette packages, and eliminating menthol in cigarettes would go a long way toward improving the health of American kids and families.” |
| Wen 2015 | “As the figure below shows, the intervention group had almost five times higher successful rate (**34%**) of smoking cessation at the end of pregnancy compared to the control group (**7%**).” | “So far, financial incentives-based intervention has been shown to be the **most** effective approach to helping these struggling women to quit smoking during pregnancy.” | “Financial incentives-based intervention have been used to help people to change behaviors including quitting smoking during pregnancy.” |
